# State of the Art in Immersive Interactive Technologies for Surgery Simulation: A Review and Prospective

**DOI:** 10.3390/bioengineering10121346

**Published:** 2023-11-23

**Authors:** Zihan Deng, Nan Xiang, Junjun Pan

**Affiliations:** 1 Department of Computing, School of Advanced Technology, Xi’an Jiaotong-Liverpool Uiversity, Suzhou 215123, China; zihan.deng21@student.xjtlu.edu.cn; 2 State Key Laboratory of Virtual Reality Technology and Systems, Beihang University, Beijing 100191, China; pan_junjun@buaa.edu.cn

**Keywords:** virtual reality, augmented reality, mixed reality, interactive technology, surgery simulation

## Abstract

Immersive technologies have thrived on a strong foundation of software and hardware, injecting vitality into medical training. This surge has witnessed numerous endeavors incorporating immersive technologies into surgery simulation for surgical skills training, with a growing number of researchers delving into this domain. Relevant experiences and patterns need to be summarized urgently to enable researchers to establish a comprehensive understanding of this field, thus promoting its continuous growth. This study provides a forward-looking perspective by reviewing the latest development of immersive interactive technologies for surgery simulation. The investigation commences from a technological standpoint, delving into the core aspects of virtual reality (VR), augmented reality (AR) and mixed reality (MR) technologies, namely, haptic rendering and tracking. Subsequently, we summarize recent work based on the categorization of minimally invasive surgery (MIS) and open surgery simulations. Finally, the study showcases the impressive performance and expansive potential of immersive technologies in surgical simulation while also discussing the current limitations. We find that the design of interaction and the choice of immersive technology in virtual surgery development should be closely related to the corresponding interactive operations in the real surgical speciality. This alignment facilitates targeted technological adaptations in the direction of greater applicability and fidelity of simulation.

## 1. Introduction

The physiological complexity and heightened risk of complications associated with surgical procedures pose significant challenges to the skillsets of surgeons. The steep learning curve for surgical skills requires substantial effort, and the traditional apprenticeship model falls short of the growing demand for talent [1]. Therefore, exploring innovative approaches to surgical skill training is crucial. Surgical simulation has been incorporated into medical curricula to enhance the surgical skills of doctors, nurses, and surgical teams, ensuring their excellence in real surgical scenarios [2,3,4,5,6,7,8]. Based on the materials employed in the simulation process, traditional simulation models can be categorized into two primary groups: synthetic models and biological models. Synthetic materials such as silicone organ models are readily available but lack visual realism. Biological models, such as cadavers and live animals, are considered better training materials, but they can potentially raise ethical and moral controversies and incur high costs in long-term usage [9,10,11]. In recent years, the rapid development of immersive technologies has made it possible to integrate them into medical training, especially in the field of surgery simulation [12,13]. We have witnessed a continual emergence of related work and research outcomes that not only broaden the applications of surgical simulation but also enhance the quality and realism of simulation techniques, making significant contributions to training surgical personnel and reducing surgical complications. Immersive interactive technologies are revolutionizing the face of surgical simulation, and an increasing number of researchers have shown significant interest in their applications in this field [7]. Therefore, to provide researchers with a rapid overview of the vibrant developments in the application of interactive technologies in this domain, it is essential to review and summarize the recent emerging achievements and the latest trends.

Immersive technologies are highly interactive approaches that immerse users in virtual environments through extensive rendering and computation. These technologies are often associated with virtual reality (VR), augmented reality (AR), and mixed reality (MR), which in recent years have been collectively referred to as extended reality (XR) [14,15]. These technologies have found applications in the field of medicine, where their interactive paradigms have injected new vitality into surgical simulation training. When it comes to immersive interaction, VR, AR, and MR all extend reality in unique ways. VR immerses users entirely in a virtual world using head-mounted displays, controllers, and sensors [14,15]. AR enhances real-world experiences by overlaying real-time information on portable wearable devices, while MR combines elements of both, enabling users to interact with virtual objects in the real world.

In VR-based surgical simulation systems, force-feedback devices are typically used as input devices for virtual interaction [4,16]. On the other hand, AR or MR-based systems, in addition to the interaction with force-feedback devices, also employ 3D-tracking methods to capture dynamic data from target objects such as surgical instruments, enabling interaction with the virtual environment [11,17]. Thus, from the foundational principles of interaction and the perspective of algorithms, this study focuses on two core aspects of technology that significantly impact immersive interactive surgical simulation: haptic rendering and tracking. The precision of surgical force application relies on haptic feedback. To better simulate tactile responses in virtual environments, a combination of controllers and haptic feedback devices is necessary [18]. Currently, haptic feedback primarily involves haptic rendering, using algorithms to approximate the physical properties of tissues during surgery for a realistic simulation. Tracking technology, especially the tracking of surgical instruments, is crucial for virtual surgical simulations as it directly impacts the user’s interactive experience. It serves as the bridge between humans, computers, and surgical instruments, requiring high precision and low latency [19]. Therefore, investigating the latest advancements in these two technologies is one of the focuses of this work.

Beyond investigating specific techniques, from a practical perspective, the selection and development of interactive technologies for virtual surgery should consider the characteristics and requirements of different types of surgeries to ensure that simulated surgical procedures and interaction means closely resemble real ones, thereby enhancing the training effectiveness and surgical skills of medical professionals. This can be achieved through customized technology development and continuous improvement to meet the needs of various surgical fields. When considering the methods and modes of surgical simulation, categorizing surgeries into minimally invasive and open surgeries based on the size of the surgical incision can provide a clearer understanding of the pros and cons of interactive technologies under different surgical characteristics [20,21]. These distinct surgical processes imply different interaction requirements for virtual surgery, likely leading to the use of different interactive techniques. Thus, discussing the performance of various surgical simulation methods based on this classification can offer researchers a preliminary insight into the diverse applications of immersive interactive technologies.

The rapid development of immersive interactive technologies has made it essential to systematically examine their applications and categorizations, as discussed earlier. Such an investigation and analysis not only help scholars gain clarity on research directions but also empower innovation in the field’s future. In prior research on immersive virtual surgery, there have been studies focusing on the application of XR technologies in clinical and surgical simulation [4,12,13,16,22,23,24]. However, they often focus on specific surgical simulation applications or the development of certain visualization techniques such as rendering and deformation algorithms, lacking reviews from the interactive technology perspective. Therefore, based on these objectives, this paper will subsequently discuss the technical details and recent advancements in haptic rendering based on force-feedback devices and surgical instrument tracking. It will investigate their impact on surgical simulation using XR devices, and will also review different immersive interaction methods’ pros and cons in various surgical specialties under the classification of minimally invasive and open surgeries. Finally, the findings of this investigation will be summarized, and prospects for opportunities and challenges in the field’s future will be explored.

## 2. Haptic Rendering

**Figure 1:**
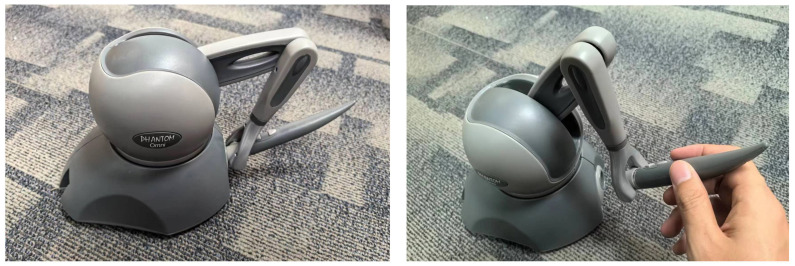
Commonly used force-feedback device (PHANTOM^®^ Omni) in virtual surgery systems (please refer to [25,26] for more details on hardware specification).

In order to enhance users’ haptic feedback and confirm the effectiveness of their actions during the human–computer interaction process, electronic devices often incorporate additional equipment to provide force or tactile feedback. In surgical procedures, medical professionals need to perceive the level of force applied during tasks such as cutting and suturing, as well as the tactile sensation of tissues to ensure the surgical procedure’s soundness. The necessity for this feedback tends to increase as surgical incisions become smaller and physiological complexities rise. Against the backdrop of an increasing emphasis on patient comfort, there is a growing demand for greater precision in surgical procedures. Therefore, the incorporation of haptic rendering technology and force-feedback devices (see Figure 1) in virtual surgical training becomes essential, allowing surgeons to better prepare for surgical interventions. It is worth noting that haptic interfaces, owing to their ability to simulate object forces and motions more effectively than tactile feedback alone, offer a wider range of applications in pure VR surgical simulation solutions. This section will primarily focus on force-feedback devices as a means of simulating tool–tissue interactions.

As a key component of computer haptic technology, haptic rendering refers to the process of computing force or tactile feedback to enable users to experience touch and perceive the virtual world [27]. From a physical standpoint, haptic rendering based on force-feedback devices is typically achieved through spring and damping models to establish virtual coupling, thereby connecting the user’s actions to virtual objects [28,29]. Specifically, the movements of force-feedback devices are converted into forces or torques acting on virtual instruments, such as surgical tools, and changes in these virtual instruments provide force or torque feedback. This approach provides a tool-like interaction style, reducing the user’s learning curve and enhancing the efficiency of simulated surgical training. More specifically, the core of simulating force-feedback device motion is the study of tool–tissue interactions. By creating physical models, computers can accurately simulate tissue deformation and generated internal forces based on the information conveyed by the device, enabling medical professionals to experience more realistic tissue characteristics.

The interaction between tools and tissues involves concepts from continuous media mechanics. According to Sarthak and Seker [30], early researchers categorized models for simulating tissue mechanics into three main classes: (1) linear elastic methods, (2) finite element (FE) methods based on nonlinear (hyperelastic) elasticity, and (3) other techniques not relying on finite element or continuous media mechanics. Within the realm of linear elasticity, commonly used standard viscoelastic models for soft tissue modeling include the Maxwell model, Kelvin model, and Voigt model [3]. However, in consideration of nonlinear and finite element methods, some researchers opt for the Neo-Hookean model and Mooney–Rivlin model to represent soft tissues. The precision of FE models is typically determined by the density of the model’s mesh, with finer meshes yielding higher accuracy at the cost of increased computation time. Nevertheless, owing to the iterative refinement of models and advancements in hardware, FE methods remain a state-of-the-art choice among biomedical scientists for modeling the interaction between surgical tools and tissues.

On the algorithmic front, researchers in the 21st century have proposed numerous solutions based on foundational principles such as computer graphics, categorized primarily by object modeling techniques and degrees of freedom (DoF) [31]. As technology in the field advanced, algorithms evolved from initially rendering points and employing 3-DoF rendering to enabling 6-DoF rendering of complex, deformable objects [32]. This advancement allows devices to simulate more intricate virtual object feedback, making precise simulations of minimally invasive surgeries and delicate procedures possible. In recent years, methods and techniques for haptic rendering interaction have encompassed tactile rendering for tool–bone (rigid), tool–soft tissue (rigid–deformable) [33], and tool–fluid interactions [32], enhanced through visual and texture processing to enhance the realism of force feedback. Decades of accumulated technological progress in haptic rendering have laid a solid theoretical foundation for the explosive emergence of immersive virtual surgery today. Numerous haptic feedback devices have rapidly developed in recent years, such as the Sensable Phantom series of haptic devices and their iterative versions like the Geomagic Touch. These devices have made significant inroads into the medical field through efficient tactile rendering [34]. However, limitations at both the software and hardware levels continue to pose challenges to the widespread adoption of immersive surgical simulation.

Based on previous research, the current rendering algorithms and modeling are built upon the foundation of classical models in elastodynamics [31]. However, real tissues manipulated during surgery often require consideration of more complex scenarios. The inherent limitations of applied physics raise challenges in simulating certain medical scenarios since scientists do not possess a complete understanding of all physical phenomena within the intricate human body systems. In a real physical environment, there is an enormous number of parameters affecting object motion, and the current computational power falls short in supporting such computational demands. Xia [31] provided a vivid illustration of the impact of bone drilling theory on the authenticity of dental surgery simulation.

Another constraint still arises from the demand for realism. Just as with visual and auditory elements, haptic feedback also needs to be updated at a certain frequency to provide a smooth and lifelike tactile experience. In the realm of vision, the refresh rate typically should not fall below 30 Hz, while haptic feedback often requires an even higher refresh rate. Previous experiments have shown that the threshold for haptic refresh rate lies between 500 Hz and 1000 Hz [35]. Furthermore, according to Laycock [36], lower update rates are better for simulating soft tissues, whereas faster update rates aid in simulating rigid materials. Higher visual refresh rates correspond to faster changes in haptic refresh rates, resulting in enhanced realism in VR surgery. Currently, various XR devices are all striving for high visual refresh rates and high resolutions as their development direction. Achieving a balance between refresh rate and processing speed within limited hardware resources is proving to be quite challenging.

## 3. Tracking

The previous section discussed the current status and challenges of haptic rendering as one of the core technologies in pure VR surgical simulation solutions. As mentioned earlier, different XR solutions have distinct technological directions due to their varying levels of interaction with the virtual world. When it comes to AR and MR technologies, the interaction with the real world necessitates researchers to consider advancements in tool tracking technology [37]. Tracking real surgical instruments and then converting the tracking data to enable interaction between real and virtual elements is bound to broaden the applicability of virtual surgical simulations, making it possible for simulation for a wider range of surgical specialties. Some research efforts have been made in this direction. For instance, Gadwe et al. [38] and Liu et al. [39] employed 2D fiducial markers to track laparoscopic ultrasound transducer and the cylindrical surgical devices, respectively. Xiang et al. [11] proposed the use of 3D trackers to obtain the motion data of real surgical scissors and forceps. The MTIID [40] introduced 3D printed clip-applying forceps combined with the Leap Motion Tracker in a cerebral aneurysm clipping simulation system. This is evidently an important direction for the future of virtual surgical interactive technology, and it is worthy of further exploration. Currently, mainstream tracking technologies can be categorized into vision-based methods and non-vision-based methods.

### 3.1. Vision-Based Tracking

Vision-based tracking methods rely on cameras or sensors to capture the position and orientation of surgical instruments. This approach can be further subdivided into two types: *Marker-based Tracking* and *Markerless Tracking*.

Marker-based Tracking: During surgical procedures, the complex environment within the camera’s field of view often poses challenges for tracking small instruments. One solution is to attach retro-reflective markers or fiducial markers to surgical instruments or patient tissues, allowing the camera to track the tools based on these markers. Typically, markers come in the form of fiducial markers [11,41] or spherical markers [42]. Fiducial markers are relatively easy to prepare, often involving printing specific shapes on paper and affixing them at predetermined locations for positioning. Spherical markers, on the other hand, reflect infrared light, which is captured by an infrared camera for tracking purposes. This approach has been replicated in some physical model surgical simulations [42]. The use of infrared technology enables markers to be detected in dimly lit environments. However, regardless of the marker type, they introduce pre-processing time to surgery and can become contaminated during the procedure leading to tracking failures, and frequent marking procedures increase the risk of cross-contamination, inadvertently adding complexity to surgery.

Markerless Tracking: One solution for markerless tracking involves capturing and modeling with stereo cameras, applying algorithms to estimate the pose of surgical instruments [43], while another approach matches 2D images from cameras with 3D or 2D models from other modalities for tracking [44]. However, both of these methods demand advanced computer vision modeling and synthesis capabilities, increasing the complexity of acquiring preoperative patient data. Additionally, both approaches require complex camera and hardware setups, posing challenges to the portability of AR/MR devices.

In recent years, with the advancement of machine learning and neural networks, especially the outstanding performance of convolutional neural networks (CNNs) in the field of computer vision, purely visual solutions for instrument tracking have seen a promising new dawn [45]. Learning-based algorithms for instrument tracking can be broadly categorized into motion-based navigation and object segmentation. In motion-based navigation, machine learning models often consist of binary classifiers. For example, support vector machine (SVM) kernels, such as those used for denoising tracking to effectively capture high-order contextual information of samples [46], and earlier appearance models based on spatial-color Gaussian mixtures (SMOG). On the other hand, neural networks play a significant role in object segmentation. Some models that excel in medical image object segmentation tasks, like Mask-RCNN and fully convolutional network (FCN)-based architectures, including U-Net, have found applications in tracking medical instruments [47,48]. However, neural networks are not without drawbacks. Firstly, the task of data collection and labeling is extensive. Secondly, neural networks typically require substantial computational resources, often sacrificing accuracy to achieve real-time tracking for surgeries. In the context of surgery simulation, this presents a notable drawback for surgeries that demand high precision.

### 3.2. Non-Vision-Based Tracking

Non-vision-based tracking methods employ sensors to monitor the posture and movements of surgical instruments. Sensors can be attached to the surgical instruments, offering a higher level of integration compared to purely visual approaches and causing minimal harm to the patients, making them suitable for minimally invasive surgical applications with smaller incisions. Typically, mechanical arms incorporate inertial measurement units (IMUs) capable of measuring the acceleration and rotation of instruments. IMUs can provide real-time posture information, often without the need for additional camera support [2]. According to Ahmad, current IMUs integrate two to three types of sensors: IMUs consisting of accelerometers and gyroscopes can provide parameters such as acceleration, angular velocity, and rotation angle, fulfilling the requirements of 4–6-degrees-of-freedom (DoF) devices [49]. However, this common two-sensor IMU may be susceptible to attitude drift, causing the tracking system to gradually deviate from the correct posture over time, potentially leading to a loss of training effectiveness. On the other hand, three-sensor IMUs include a magnetometer, allowing for the calculation of yaw angle rotation and calibration of posture drift when combined with gyroscopes. Consequently, three-sensor IMUs can provide nine DoF for tracking surgical tools and are considered a possible future direction [49]. The inclusion of a magnetometer enhances tracking accuracy, but it can also be easily influenced by metal objects and electromagnetic fields in the surgical environment, which are often present in surgical instruments and implants made of metal. Excessive noise can not only negate the advantages of posture calibration but also affect its overall accuracy. Furthermore, compared to visually based approaches, purely sensor-based solutions often exhibit lower reliability and accuracy. In certain low-light and confined spatial scenarios, such as minimally invasive surgery, relying solely on visual methods can lead to performance degradation [50,51]. Therefore, integrating sensors with other modalities presents a viable approach. Huang et al. [50] proposed a mixed tracking system that combines and IMU instrument with a camera system, demonstrating the sophistication of this hybrid solution.

## 4. Minimally Invasive Surgery Simulation

In Section 2 and Section 3, the survey embarked on a technical perspective, unveiling the current state of core technological developments in various immersive interactive technologies for surgery simulation. It becomes evident that the technological underpinnings required for VR, AR, and MR differ significantly. However, it is imperative to underscore that, to ensure customization and adaptability in surgical simulation, the choice and optimization of interactive technologies used in virtual surgical simulations should align with the specific surgical procedure. This alignment is crucial for simulating the intricacies and nuances of real surgical procedures. Based on distinct surgical paradigms, this section and the following one will separately summarize and delve into the developments and merits of immersive interactive technologies in the context of minimally invasive surgery (MIS) and open surgery simulation.

According to Stanford Medicine [20], surgeries can be categorized into two types based on the size of the incision: open surgery and minimally invasive surgery (MIS). Open surgery aligns more closely with the conventional perception of surgical procedures, involving the cutting of a patient’s skin or tissues to facilitate comprehensive observation of relevant structures or organs. In contrast, MIS generally encompasses techniques that do not necessitate extensive incisions, primarily involving procedures such as laparoscopy or endoscopy and similar modalities. Compared to traditional open surgery, MIS offers several advantages, including smaller incisions, faster recovery times, and fewer postoperative complications. It is typically performed by introducing fine, long-handled surgical instruments through small incisions to access the patient’s internal structures. Surgeons monitor the surgical process through high-resolution displays, often facilitated by cameras and fiber optic transmission technology, which transmits in real time the surgical scene to screens. This mode of operation requires surgeons to perform surgery on a virtual screen and relies on visual and haptic feedback for precision during intricate procedures [52]. The distinctive characteristics of minimally invasive surgery have elevated its significance in the field of surgical medicine, where complexity and precision are paramount.

**Figure 2:**
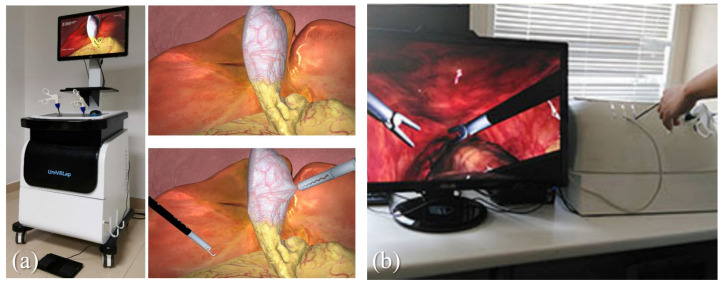
Exemplary MIS simulation systems. (**a**) illustrates a virtual reality cholecystectomy simulator with a complete movable worktable [54]. (**b**) demonstrates a laparoscopic simulator with high-fidelity soft tissue rendering [55].

The applications of minimally invasive surgery are extensive, and nowadays, surgeons employ MIS for diagnosing and treating a variety of cancers and significant medical conditions [53]. MIS is most commonly used to examine or operate on various cavities within the body, such as the digestive tract, respiratory tract, thoracic cavity, and reproductive system. In recent years, there has been a plethora of immersive surgical simulation work focusing on these types of surgical procedures (see Figure 2).

Table 1 presents some representative works on immersive interactive simulation technologies for various MIS procedures. Through a thorough review and collection of internet databases, this body of work encompasses a wide array of aspects, including the creation of virtual surgical environments, the assessment of surgical training modes, and systematic reviews within specialized domains. A comprehensive analysis reveals that minimally invasive surgery simulations primarily focus on laparoscopic and endoscopic procedures. Laparoscopy typically involves surgical procedures through small abdominal incisions, while endoscopy utilizes natural orifices such as the mouth for access. These techniques find widespread applications in examining and operating on virtually all major internal organs, including those in the digestive and respiratory systems, making research in this area indispensable.

It is noteworthy that the majority of immersive simulations for minimally invasive surgery are based on virtual reality (VR) systems. This preference is largely attributed to the maturity of endoscopy simulation technology, and the interaction mode in VR systems (hands–controllers–display screen/goggles) can effectively replicate the interaction in real MIS procedures (hands–graspers–display screen). According to surveys, the experience with augmented reality (AR) devices tailored for laparoscopy and endoscopy simulations remains limited. Viglialoro’s research [37], as of the publication of the study, identifies ProMIS as the sole commercial device available, which demonstrates satisfactory performance in AR-based MIS surgical simulations. Botden et al. [56] validated newly developed metrics for assessing laparoscopic suturing techniques on the ProMIS augmented reality simulator. Although this study illustrates the usefulness of AR in MIS simulation as well, its timeliness and too few similar studies have exposed the limitations. In contrast, on the VR side, the similarity of interaction patterns and the more optional devices also allow more VR-based MIS simulation to be practiced [54,63,66,67].

One of the important aspects of surgery simulation is to implement the user’s interactive behavior with the virtual environment. Endoscopic procedures often rely on mechanical arms and surgical instruments for manipulation, which is very similar to operating force-feedback devices based on long-handled controls. Consequently, a significant portion of research endeavors focuses on integrating haptic feedback devices. On the other hand, upon investigation, it can be found that AR tends not to integrate better force-feedback devices, but targeted improvement or evaluation in the visual aspect [37,56]. In a word, compared to the advantages of VR in MIS simulation, AR or MR systems do not appear to be the primary choice for MIS simulation at present.

## 5. Open Surgery Simulation

Open surgery and minimally invasive surgery exhibit significant differences in their methods of operation and technical requirements. Open surgery necessitates larger incisions, providing surgeons with a more direct field of view, facilitating easy observation of the patient’s internal organs or tissues. In contrast to the typical visibility of the surgical site in open procedures, particularly for delicate areas such as the nervous system or the eyes, surgeons often employ microscopy instruments to perform more precise maneuvers. Some critical surgeries in this category involve procedures like brain surgery, liver tumor excision, and implant placements.

Regarding the choice of instruments, open surgery diverges from the standardized mechanical arms and catheters used in MIS, as it employs a diverse range of surgical instruments capable of executing more complex tasks. For instance, general open surgery may involve various-sized scalpels, forceps, and sutures, whereas microsurgery demands even finer surgical instruments. Undoubtedly, the unique characteristics of open surgery, distinct from MIS, underscore the distinct requirements for surgical simulation and interactive technologies, warranting an investigation into previous immersive interactive technology-related research in this field.

**Figure 3:**
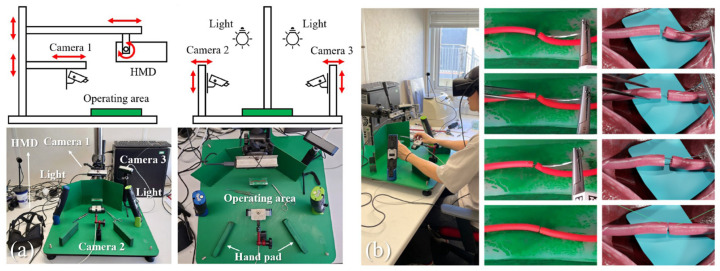
A mixed-reality-based prototype system for micro-anastomosis [11]. (**a**) depicts the workbench design of the system. The white arrows indicate various components in the workbench, including a head-mounted-display (HMD), several camera sensors, etc. (**b**) shows a simulation process of the microvascular hepatic artery reconstruction (MHAR) surgery by using the system.

Similarly, over the past decade, some representative works in the field of open surgery simulation are listed in Table 2. Figure 3 shows the design and use of an MR-based surgery simulation system. In the domain of open surgery simulation, AR and MR have taken center stage. Given the alignment of technical requirements and commercial demands for open surgery, the market has seen the emergence of numerous established AR/MR hardware developers offering a range of devices for research and development in surgical simulation, thereby establishing a robust hardware foundation. Compared to MIS simulation, more emphasis has been placed on tracking technologies within the technological core for open surgery simulation. Some notable research studies in using common AR and MR devices have introduced novel techniques, in dentistry, tumor treatment, and orthopedics, respectively [68,69,70]. Some prior studies [17,71,72,73,74] have contributed various perspectives on tracking technologies. Emerging technologies such as ultrasound have further empowered AR and MR to excel in open surgery [17]. Open surgery typically demands meticulous attention to various patient parameters [75], increasing the cognitive load on surgeons. The research in AR and MR aims to provide more realistic and richer data from the user’s perspective, simplifying information retrieval in surgical simulation and allowing surgeons to focus on operation aspects, thereby promoting more precise simulations.

Some open surgery simulation research also incorporated VR technologies [76,77,78]. One advantage of these studies is the ability to provide a fully immersive environment for surgical training. However, considering that the surgical environment in open surgery typically involves more physical variables, visual feedback becomes more challenging. From a technical standpoint, as mentioned earlier, compared to AR and MR, pure VR solutions require reliance on haptic devices to interact with virtual objects, while the simulation details for open surgery are significantly more intricate than MIS, which could be in a purely virtual environment. Therefore, researchers in the field of open surgery simulation may be more inclined towards enhancing reality rather than building pure virtual worlds.

Overall, the current three main immersive technologies have made significant contributions to virtual surgery simulation. Simulations of different surgical types tend to favor different technological choices based on their operating modes and interaction characteristics. MIS simulation often leans towards pure virtual technology based on VR, while open surgery simulation tends to select AR or MR technology based on the surgical specialties.

**Table 2:** Overview of Highlighted Work on Immersive Interactive Technologies for Open Surgery Simulation.

Author and Year	Surgical Procedure	Immersive Interaction Type	Description	Device and/or Method
Lin et al., 2015 [68]	Dental implant	AR	Development of an augmented reality-based dental implant simulation system	Sony^®^ HMZ-T1 personal 3D viewer, CCD and marker tracking
Watanabe et al., 2016 [69]	Tumor resection surgery	AR	An augmented reality-based navigation system was developed to realize the full tracking function by overlaying MRI and CT images	Tablet and VICON^®^ tracking system with 6 cameras
Yoon et al., 2017 [75]	Parieto-occipital ventriculoperitoneal shunt placement (Ventricular Catheter Placement)	AR	Practice of surgical assistance via a wearable flat screen monitor mounted on a magnifying glass	Google^®^ glasses
Weidert et al., 2019 [71]	Distal Interlocking	AR	Evaluating the Feasibility of a Video-Augmented Fluoroscopy (VAF) Technique for Distal Interlocking of Intramedullary Nails Using a Camera-Enhanced Mobile C-Arm (CamC)	Marker-based tracking and video-augmented instrument tracking
Coelho et al., 2020 [72]	Metopic craniosynostosis	AR	Development of a preoperative planning method combining hybrid modeling and augmented reality (AR) for correction of deviated cephalic deformity	Cell phones with AR applications and vision tracking method
Golse et al., 2021 [73]	Liver section surgery	AR	Practice of hepatic resection by markerless visual tracking technique	Markerless tracking and 3D-CT scanning
Fushima and Kobayashi 2016 [74]	Orthognathic	MR	Presentation and evaluation of a mandibular motion tracking system	Three-dimensional computed tomography and device tracking technology
Ameri et al., 2017 [17]	Internal jugular vein cannulation	MR	Development of a mixed reality ultrasound guidance system tailored to central line insertions	Ultrasound-assisted visual tracking
McJunkin et al., 2018 [70]	Lateral skull base anatomy	MR	An MR device-based system was developed for three-dimensional (3D) visualization of interactive holograms fixed at specific points in physical space for lateral skull base dissection	Microsoft^®^ HoloLens and marker-based vision tracking method
Xiang et al., 2023 [11]	Microvascular anastomosis	MR	A vision-based tracking system is proposed to simultaneously track surgical instruments and artificial blood vessels	TsFPS [79] based high accuracy surgical instrument tracking
Alaraj et al., 2015 [76]	Aneurysm clipping surgery	VR	Development of a real-time sensory haptic feedback virtual reality aneurysm clipping simulator	Immersive Touch^®^ platform with haptic feedback technique
Azarnoush et al., 2015 [77]	Tumor section surgery	VR	Evaluating the Effectiveness of Metrics Extracted from the NeuroTouch Platform for Brain Tumor Surgery	NeuroTouch Platform with haptic feedback
Pulijala et al., 2018 [78]	Orthognathic surgery	VR	Development and validation of a novel immersive virtual reality (iVR)-based Le Fort I osteotomy training tool based on Oculus^®^ Rift and Leap^®^ Motion devices	Oculus^®^ Rift and Leap^®^ Motion VR platform

## 6. Discussion

Surgery simulation plays a crucial role in training medical professionals. To facilitate a comprehensive understanding of the latest developments in immersive interactive technologies for surgery simulation across various fields, it is necessary to investigate and discuss these advancements. Through a comprehensive review, we can assert that within the current landscape, the three branches of immersive interactive technologies, namely virtual reality (VR), augmented reality (AR), and mixed reality (MR), have all played pivotal roles in medical training and enhancing surgical skills.

Several foundational technologies are closely related to the development of immersive interactive technologies. Haptic rendering, as an integral part of immersive interactive technologies, empowers the simulation of tool–tissue interactions in virtual environments. It is typically deployed in VR-based surgical simulation systems. On the other hand, tracking technologies are crucial to AR and MR-based surgical simulation systems. Although vision-based tracking methods have become quite mature and have been applied into virtual surgeries, the integration of vision and non-vision solutions may be considered as a trend to enhance precision and interactivity.

Surgical procedures encompass a wide range of medical fields and classifications. Based on incision size, they can be categorized as minimally invasive surgery (MIS) and open surgery. Minimally invasive surgeries are primarily observed from a camera perspective and involve interaction through multifunctional channels and robotic arms, making them suitable for simulation using VR technology with haptic feedback devices. On the contrary, open surgeries are characterized by larger incisions and complex interactive environments, aligning more closely with AR and MR technologies. After investigating the technology and applications, it is evident that immersive interactive technologies have evolved towards a multimodal approach capable of simulating various types of surgeries. However, the precision requirements of different surgical procedures still demand varying technological support to cater to the diverse skill levels and training needs of medical professionals. Thus, the critical selection of appropriate immersive interactive technologies based on the actual surgical type and approach remains of paramount importance. This choice aids in improving training efficacy, reducing risks, and enhancing the overall performance.

### 6.1. Challenges

During our investigation, we found that there are still some issues in current immersive interactive technologies, including the need for improvement in force feedback and tracking accuracy, the exploration of multimodal fusion, and the development of frameworks or toolkits. These issues also pose challenges for the development of software and hardware. Haptic rendering and tracking technologies have yet to achieve true high fidelity. Algorithms for basic physics models have recently encountered optimization bottlenecks, and the interaction between tools and tissues, as well as the physical models of elasticity, still require improvement. Considering tracking technologies, the deep learning-based approach needs to improve its generality, while traditional marker-based localization may raise concerns about resource wastage and biological contamination.

From a hardware perspective, although current hardware capabilities allow us to compute and simulate some surgical procedures, issues such as high costs, limited portability, and platform interoperability persist. Current common VR-based surgical simulation devices are bulky and come at a high cost [54,63,76]. These limitations hinder the advantages of immersive technology training, particularly in regions already lacking in medical resources. Moreover, commercialization remains elusive. The current supply chain’s ability to support the widespread availability of high-quality, high-performance devices for surgical simulation training is still in question. Lastly, the multitude of devices in the market often lacks a unified industry standard, making device and system compatibility one of the limiting factors for development.

### 6.2. Future Prospect

In recent years, the rapid development and application of XR-related technologies have brought about a transformation in the field of immersive surgical simulation. The anticipation is high for new virtual surgery systems with improved realism, higher precision, and more standardized development processes. As aforementioned, different immersive interactive technologies need to be considered for surgeries with varying characteristics. However, as the boundaries between future XR technologies are expected to blur, synergies between VR, AR, and MR can be a trend [14]. On the hardware and software fronts, more and more developers are likely to offer more powerful XR devices and a choice of technologies, proposing more realistic biomechanical simulations, physiological responses, and tool–tissue interactions. Whether it is VR, AR, or MR technology, exploring optimization through enhanced algorithms, multimodal fusion, and the integration of deep learning methods may offer feasible pathways for improvement. Furthermore, with the widespread adoption of 5G and artificial intelligence, immersive interactive technologies are also expected to benefit from these advancements [80], offering lower signal latency, faster algorithms, higher precision, and clearer visuals, along with multi-faceted AI assessment of training outcomes, which are expected to drive further development in the field of virtual surgery simulation. From the perspective of the supply chain and market, device manufacturers should consider integrating and upgrading the entire supply chain to continually enhance the performance and quality of head-mounted displays, sensors, haptic feedback devices, and tracking systems while reducing costs to facilitate wider adoption. Finally, both software and hardware developers should advocate for industry standardization to promote software–hardware integration.

## Figures and Tables

**Table 1:** Overview of Highlighted Work on Immersive Interactive Technologies for Minimally Invasive Surgery Simulation.

Author and Year	Surgical Procedure	Immersive Interaction Type	Description	Device and/or Method
Botden et al., 2009 [56]	Laparoscopic surgery	AR	Validation of newly developed laparoscopic suturing on the ProMIS augmented reality simulator	ProMIS v2.0 augmented reality (AR) simulator with visual tracking solution
Viglialoro et al., 2019 [37]	Laparoscopic cholecystectomy	AR	Reported results of the long-term development phase of a hybrid simulator for laparoscopic cholecystectomy	Electromagnetic emitter/visual tracking-based AR device
Zátonyi et al., 2005 [57]	Hysteroscopic surgery	VR	A state-of-the-art graphical blood flow simulation designed to meet the specific requirements of virtual hysteroscopic surgical bleeding simulation	3D Fluid Simulation
von WebSky et al., 2012 [58]	Laparoscopic surgery	VR	Compiled a set of criteria to demonstrate the performance and feasibility of the Simbionix LAP Mentor for basic laparoscopic training for novice surgeons	Simbionix^®^ LAP Mentor with haptic feedback device
Cohen et al., 2013 [59]	Endoscopic neurosurgery	VR	A review of basic concepts and applications in endoscopic neurosurgery training	Unspecified haptic feedback device
Breimer et al., 2017 [60]	Endoscopic neurosurgery	VR	Evaluation of the Educational Benefits of Virtual Reality (VR) and Physical Simulation Models for Endoscopic Third Ventriculostomy (ETV)	PHANTOM^®^ Omni (haptic feedback device) and NeuroTouch
Qian et al., 2017 [61]	Laparoscopic surgery	VR	Proposed a set of tailored key technologies for laparoscopic surgery simulation	Unspecified haptic feedback device
Matsuo et al., 2018 [62]	Endoscopically assisted implant surgery	VR	Established a system of endoscopically assisted VR for implant surgery using a head-mounted display	Sony^®^ HMS-3000MT and Olympus 4mm nasoscope
Frederiksen et al., 2019 [63]	Laparoscopic surgery	VR	Cognitive load of immersive VR laparoscopic simulation of surgery was assessed	Simball 4D joysticks (haptic feedback device) and Oculus Rift
Aoki et al., 2020 [64]	Laparoscopic distal pancreatectomy	VR	Evaluate the impact of 3DVE guidance in laparoscopic distal pancreatectomy (LDP)	SYNAPSE VINCENT Volume Analyzer
Pan et al., 2020 [54]	Laparoscopic cholecystectomy surgery	VR	A VR simulation framework based on PBD for cholecystectomy that has been applied to laparoscopic cholecystectomy training in several hospitals	Unspecified haptic feedback device
Tai et al., 2021 [65]	Thoracoscopic surgery	VR, AR	Presented the AR visual rendering and haptic modeling to study the potential benefits of thoracoscope surgical skills	Unspecified haptic feedback device
Lohre et al., 2020 [66]	Spinal endoscopic surgery	VR, AR, MR	Reviewed work on endoscopic spinal surgery	Multiple works on different devices are reviewed, such as H3D and Volume Haptics Toolkit, PHANTOM^®^ haptic device graphical user interface and various unspecified haptic devices
